# Cultivating Resilience in Dryland Soils: An Assisted Migration Approach to Biological Soil Crust Restoration

**DOI:** 10.3390/microorganisms11102570

**Published:** 2023-10-15

**Authors:** Sierra D. Jech, Natalie Day, Nichole N. Barger, Anita Antoninka, Matthew A. Bowker, Sasha Reed, Colin Tucker

**Affiliations:** 1 Department of Ecology and Evolutionary Biology, University of Colorado Boulder, Boulder, CO 80309, USA; 2 Colorado Water Science Center, U.S. Geological Survey, Grand Junction, CO 81506, USA; 3 School of Forestry, Northern Arizona University, Flagstaff, AZ 86001, USA; 4 Center for Ecosystem Science and Society, Northern Arizona University, Flagstaff, AZ 86001, USA; 5 Southwest Biological Science Center, U.S. Geological Survey, Moab, UT 84532, USA; 6 Manti-La Sal National Forest, U.S. Forest Service, Monticello, UT 84535, USA

**Keywords:** soil degradation, climate change, restoration, microbial ecology, assisted migration, biological soil crust, cyanobacteria

## Abstract

Land use practices and climate change have driven substantial soil degradation across global drylands, impacting ecosystem functions and human livelihoods. Biological soil crusts, a common feature of dryland ecosystems, are under extensive exploration for their potential to restore the stability and fertility of degraded soils through the development of inoculants. However, stressful abiotic conditions often result in the failure of inoculation-based restoration in the field and may hinder the long-term success of biocrust restoration efforts. Taking an assisted migration approach, we cultivated biocrust inocula sourced from multiple hot-adapted sites (Mojave and Sonoran Deserts) in an outdoor facility at a cool desert site (Colorado Plateau). In addition to cultivating inoculum from each site, we created an inoculum mixture of biocrust from the Mojave Desert, Sonoran Desert, and Colorado Plateau. We then applied two habitat amelioration treatments to the cultivation site (growth substrate and shading) to enhance soil stability and water availability and reduce UV stress. Using marker gene sequencing, we found that the cultivated mixed inoculum comprised both local- and hot-adapted cyanobacteria at the end of cultivation but had similar cyanobacterial richness as each unmixed inoculum. All cultivated inocula had more cyanobacterial 16S rRNA gene copies and higher cyanobacterial richness when cultivated with a growth substrate and shade. Our work shows that it is possible to field cultivate biocrust inocula sourced from different deserts, but that community composition shifts toward that of the cultivation site unless habitat amelioration is employed. Future assessments of the function of a mixed inoculum in restoration and its resilience in the face of abiotic stressors are needed to determine the relative benefit of assisted migration compared to the challenges and risks of this approach.

## 1. Introduction

Drylands are water-limited ecosystems, such as deserts and rangelands, that cover ~40% of the global terrestrial surface and support 38% of the global human population [1]. Drylands are expected to expand globally within the next century due to land use practices (e.g., overgrazing) and climate change [2,3]. These drivers have also resulted in widespread soil degradation, which impacts critical ecosystem functions like plant productivity, the stability and fertility of soils, and resistance to future disturbance [4]. Soil degradation in drylands can have profound impacts on human livelihoods and human health [5,6]. The interactive effect of soil degradation and climate change makes reversing degradation extremely challenging [7,8]. Biological soil crusts (biocrusts), communities of mosses, lichens, cyanobacteria, and other organisms that grow at the soil surface [9], provide valuable ecosystem services in drylands [10,11,12,13] but are vulnerable to both physical disturbance and climate change [14,15,16,17]. Changes to biocrust composition or function can be slow to recover following disturbance [18], even with active restoration interventions [19].

Physical disturbance and climate change can result in the abrupt loss of late-successional biocrust organisms like moss, lichen, and dark-pigmented cyanobacteria [14,20] and the high rates of carbon fixation, nitrogen fixation, and soil stabilization that these organisms provide [21,22]. Early-successional cyanobacteria may be more persistent or better able to reestablish degraded soils following disturbance [17,23,24], which helps to maintain some critical ecosystem functions, albeit at reduced levels. In addition, cyanobacteria have fast generation times under favorable conditions [25], are tolerant to UV exposure [26] and cycles of hydration and desiccation [27]. In addition to these cyanobacterial contributions, recent work has shown that biocrust heterotrophs play important roles in the community, including for biocrust formation [28]. Biocrust communities (both autotrophs and heterotrophs) may be cultivated as inocula in the laboratory [25,29] or greenhouse [30,31]; however, in order to produce inoculum at a scale relevant for landscape restoration, field cultivation may be necessary. Outdoor growth could also help prepare the inoculum community for establishment, making them more successful in the face of real-world temperature, moisture, and light conditions. Under challenging conditions, multiple restoration strategies can be used in conjunction with inoculation (e.g., erosion control) to enhance the cultivation and establishment of biocrusts [32,33]. 

Biocrust cyanobacteria vary in taxonomic composition with climate. For example, *Microcoleus vaginatus* is widespread but tends to be more abundant in drylands with lower mean annual temperature [34]. The *Microcoleus steenstrupii* complex, now known as the Coleofasciculaceae family, includes *Crassifilum* spp. and *Parifilum* spp., which are more abundant with higher mean annual temperature [35]. These temperature preferences for biocrust cyanobacteria are particularly interesting in a “prestoration” framework for restoration, which aims to improve restoration effectiveness by including taxa in restoration that are tolerant to not only current but also future climate conditions [36]. For example, on the Colorado Plateau, temperatures may increase ~3 °C degrees by 2050, precipitation may become more variable, and drought may be more frequent [37,38]. Furthermore, climate on the year of restoration implementation can have a strong influence on the outcome of restoration [39]. Therefore, it is worth investigating the value of including both locally adapted and hot-adapted organisms in a restoration inoculum mixture. This bet-hedging strategy has been employed in plant-based restoration efforts in drylands and yielded varying outcomes [40,41,42]. Assisted migration, the moving of organisms from climates more likely to match the expected climate for a location, is one way in which we might incorporate hot-adapted organisms into cool-desert restoration [43]. It has been suggested that biocrusts are limited in their climate plasticity; however, it may be possible to move mosses and lichens to milder climates than their habitat of origin (e.g., moving organisms to higher elevations) [44]. It is not yet known how hot-adapted microbial communities respond to cool desert conditions (like that of the Colorado Plateau). 

In this study, we ask how local and hot-adapted biocrust cyanobacteria respond to field cultivation at a cool desert site when grown separately and as a mixture. Second, we ask how biocrust cyanobacteria respond to habitat amelioration (growth substrate and shade) during field cultivation. To address these questions, we cultivated biocrust inocula containing moss, lichen, cyanobacteria, and heterotrophic microorganisms at an outdoor facility and used 16S marker gene sequencing and gene abundances to compare the cyanobacterial communities of the inocula to one another and to in situ reference biocrust communities. On degraded soils, a growth substrate provides the biocrust organisms with a stable surface while the shade cloth can prevent the loss of propagules due to wind and water erosion. From a climate amelioration perspective, the shade cloth also reduces UV exposure, traps moisture, and reduces daytime temperature. We predicted that hot-desert cyanobacteria would survive cultivation in a cool desert and that the growth substrate and shade cloth would support cyanobacterial community growth, given that these biocrust organisms were continuously exposed to stressful field conditions throughout cultivation. We foreground results from the cyanobacterial response but recognize that whole biocrust communities were cultivated in this project.

## 2. Materials and Methods

### 2.1. Biocrust Collection 

**Figure 1:**
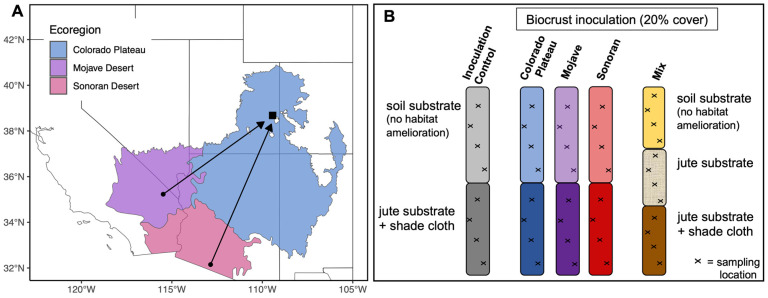
(**A**) Map of biocrust collection sites. Square point represents both the Colorado Plateau collection locations and the cultivation facility. Black arrows represent the movement of hot desert biocrusts to the Colorado Plateau. The blue, purple, and pink polygons represent the footprints of the Colorado Plateau, Mojave, and Sonoran Deserts, respectively. Modified from Young et al. (2016) [43] (with permission). (**B**) Experimental set-up at the cultivation facility, with columns corresponding to different types of biocrust inoculum (e.g., Colorado Plateau, Mojave, Sonoran) and sections of each column corresponding to habitat amelioration treatments (e.g., soil or jute substrate). Note that the intermediate jute substrate treatment was only evaluated with mixed inoculum.

We collected biocrust inocula from three deserts in the United States: two locations in the Colorado Plateau (38.719357, −109.554302 and 38.545039, −109.525265), one location in the Mojave Desert (35.710159, −115.300441), and one location in the Sonoran Desert (32.971248, −112.664660) (Figure 1A & Table 1) in spring and summer of 2018. We scraped intact biocrusts from the soil surface down to approximately 1 cm using hand tools and stored the material dry, indoors, and in the dark for 2–6 months until application at the cultivation facility in the Colorado Plateau. Some soil microorganisms are viable for up to ten years in stockpiled topsoil [45]. Harvested biocrusts were salvaged from sites that were scheduled for destructive future land uses so that the large amount of harvested biocrust inoculum did not represent a disturbance to an otherwise intact ecosystem [46]. In early December 2020, we returned to the Sonoran and Mojave collection sites, and in May 2021, we returned to the Colorado Plateau sampling sites to obtain samples of the initial biocrust communities for inclusion in the molecular analyses. At each site, we randomly sampled ten 1 cm^3^ cores across a 10 m^2^ area containing biocrust communities representative of the initial collection effort. We pooled these cores and stored them at −20 °C in sterile Whirl-Pak (Chicago, IL, USA) bags until laboratory analysis.

### 2.2. Biocrust Cultivation

To produce a large quantity of biocrust inoculum for future restoration efforts, we cultivated biocrust inoculum at the Mayberry Native Plant Propagation Center in Moab, UT (38.682987, −109.428330). This cultivation facility is at an elevation of 1235 m with Entisols (Thoroughfare series [47]) that had loam to silt loam texture as determined in the lab following standard methods [48]. The average annual precipitation is 29 cm, and average annual temperatures range from 6 to 20 °C. During the 12-month cultivation period, 7 months had mean monthly temperatures below the 30-year normal, with May 2019 reaching 6 °C below the May normal (17.4 °C). Five months had mean monthly temperatures above the 30-year normal, reaching 3 °C above normal (20.9 °C) in September 2019. Overall, precipitation was higher than the 30-year normal during the cultivation period, with seven months above normal, ranging from 0.5 to 8 cm above normal. For five months, precipitation was below normal, ranging from 2 to 2.4 cm below normal (Supplementary Figure S1, climate data from the Western Regional Climate Center (WRCC), wrcc.dri.edu (accessed February 2023), Castle Valley 1SE Station). 

In fall 2018, rows were cleared of vegetation and prepared for biocrust cultivation. We prepared cultivation rows (100 m × 1.5 m) by scraping to a depth of ~2 cm using a skid-steer loader to remove all vascular plants and create a level cultivation surface. We piled the scraped soils in parallel rows between each cultivation row. In October 2018, we spread the harvested biocrusts across the soil surface at a rate equal to 20% cover of the original harvest, and two irrigation hoses were laid parallel to the rows to deliver Colorado River water during the spring and fall only at two watering regimes: (A) 1.5 h per day for three days per week and (B) 3 h per day once per week. We showed that communities cultivated under the two watering regimes were not different, so samples from these watering regimes were analyzed together. In addition, the addition of water was meant to increase the growth of the inoculum and is not meant to be a realistic precipitation regime. We cultivated biocrusts from each desert separately and together in equal proportions as a mixed inoculum. In addition, we provided two types of habitat amelioration: (1) jute substrate only (jute substrate) and (2) jute substrate with a white plastic shade cloth (jute substrate + shade cloth) and compared the two to a non-ameliorated soil surface (soil substrate). The jute was an untreated biodegradable burlap fabric (DeWitt, Sikeston, MO, USA) meant to provide a stable surface for biocrust organisms to attach to. The jute was laid directly on the soil surface, and biocrusts were inoculated on top. The white shade cloth was a UV-stabilized polyethylene with sewn edges (DeWitt, Sikeston, MO, USA), which provided shade to reduce UV exposure by 50% and also increased hydration periods by trapping moisture and reducing surface temperatures. The shade cloth was applied directly on top of the biocrust propagules and attached using soil staples (Supplementary Figure S2). Finally, rows which did not receive biocrust propagules were used as experimental controls (inoculation control). 

### 2.3. Sampling Cultivated Biocrust

One year after cultivation (October 2019), we sampled cultivated inocula by coring four samples from each cultivation row; all rows were 2 m apart, with two samples close to the center of the row and two samples near the row edge (Figure 1B), avoiding areas with mosses or lichens. Each sample was composed of seven 1 cm^3^ cores (1 cm depth), which were pooled together into sterile Whirl-Pak bags. Because there were multiple cultivation rows for each inoculum type, we selected a subset of samples using a random number generator. We included four replicates of each Colorado Plateau, Mojave, and Sonoran inocula under watering regime A (soil substrate and jute + shade treatments) and six replicates for the mixed inocula. We included six replicates of each Colorado Plateau, Mojave, Sonoran, and mixed rows under watering regime B (soil substrate and jute + shade treatments). We included four replicates for each inoculation control. This was a total of 78 cultivated samples. With the additional 12 samples from the initial biocrust communities (three replicates for each) and four extraction blanks, we had a total of 94 samples. The biocrust samples were air dried and then stored at −20 °C until laboratory analysis.

### 2.4. Molecular Methods and Bioinformatic

We homogenized the biocrust samples by grinding gently with a 70% ethanol-sterilized mortar and pestle for 1 min. We then extracted total DNA from a 0.25 g sub-sample of each sample and four extraction blanks using the Qiagen DNeasy PowerSoil DNA isolation kit (Qiagen, Germantown, MD, USA) following the manufacturer’s instructions (with one minor modification); we heated Solution C1 (65 °C) in the bead tube for 10 min prior to the bead beating step. Next, we amplified the V4-V5 region of the 16S rRNA gene with 515F (GTGYCAGCMGCCGCGGTAA) and 806R (GGACTACNVGGGTWTCTAAT) primers [49,50] with unique 12 base pair barcodes and Illumina sequencing adapters in duplicate for each sample and extraction blank and one no-template control. Our PCR reaction included 10.5 µL of PCR water, 0.5 µL of each primer at 10 µM, 1 µL of genomic DNA, and 12.5 µL of Platinum II Hot Start PCR Master Mix (Thermo Fisher Scientific, Waltham, MA, USA), and the following PCR conditions were used: 94 °C for 2 min, then 35 cycles of 94 °C (15 s), 60 °C (1 min), and 68 °C (1 min) with a final extension at 72 °C (10 min). We verified the amplification with gel electrophoresis and then cleaned and normalized the amplicons using the SequalPrep Normalization Plate (Thermo Fisher Scientific, Waltham, MA, USA) following the manufacturer’s instructions; then, we pooled the duplicates. The University of Colorado Center for Microbial Exploration performed the sequencing on the Illumina MiSeq platform (v2 300 cycle kit) (https://www.illumina.com/systems/sequencing-platforms/miseq.html). 

The absolute abundances of the 16S rRNA genes were determined via a quantitative polymerase chain reaction (qPCR) using 1.25 µL of 10 µM 515F primer (5′-GTGCCAGCMGCCGCGGTA-3′) and 1.25 µL of 10 µM 806R primer (5′-GGACTACHVGGGTWTCTAAT-3′) (IDT, Coralville, IA, USA), 5 µL of PCR water, 12.5 µL of Absolute QPCR SYBR Green Mix (2×) (Thermo Fisher Scientific, Waltham, MA, USA), and 5 µL of genomic DNA in a 25 µL reaction. Standards of *E. coli* genomic DNA were quantified using the Quanti-iT PicoGreen dsDNA Assay Kit (Thermo Fisher Scientific, Waltham, MA, USA) following the manufacturer’s instructions. We ran each sample in duplicate with a seven-point serial dilution standard curve using the CFX Connect Real-Time PCR Detection System (BioRad Laboratories, Hercules, CA, USA) and the following PCR conditions: 95 °C (15 min), followed by 40 cycles of 94 °C (45 s), 50 °C (60 s), and 72 °C (90 s), and finally, a hold at 72 °C (10 min). 

Next, we followed the DADA2 bioinformatic pipeline (version 1.22.0, https://benjjneb.github.io/dada2/) (accessed March 2022) [51,52]. First, we demultiplexed the reads (idemp, https://github.com/yhwu/idemp) with adapters and primers removed (cutadapt, v1.8.1, with default settings, https://cutadapt.readthedocs.io/en/stable/) [53]. We trimmed the forward reads (truncLen) to 145 base pairs and reverse reads to 140 base pairs using the default parameter for the error rate and a quality score threshold of 11 (trunQ). Following these filtering parameters, a mean of 88% of reads remained. Next, we dereplicated the reads, merged paired ends, and assigned amplicon sequence variants (ASVs). We removed chimeras; 99.3% of reads were non-chimeric. We assigned taxonomy to each ASV using the SILVA reference database (v138.1) (https://www.arb-silva.de/) [54]. Demultiplexed raw reads from each sample were deposited to the National Center for Biotechnology Information (NCBI) under BioProject PRJNA1025292. Individual sample accessions are listed in Table S8. 

We used phyloseq (v1.42.0) (https://joey711.github.io/phyloseq/) [55] in R (v4.2.1, https://cran.rstudio.com/) with RStudio (https://posit.co/download/rstudio-desktop/) to view and edit the ASV table. We removed ASVs without a phylum assignment and removed 425 chloroplast and 3020 mitochondria ASVs from the table (there were no eukaryotic ASVs to remove). This resulted in an average of 17,844 reads per sample (range 4610–48,634 reads) and 1.9 million reads total. We checked blank samples for contamination. We found fewer reads in the blanks than in the samples (average of 2160 reads) and no consistent pattern of contamination at the phylum level across blank samples; thus, we did not remove contaminants from the samples. 

We reassigned taxonomy for all cyanobacterial ASVs with a relative abundance greater than 0.1% in any sample using Cydrasil [56] (https://www.cydrasil.org) (accessed March 2023) and iTol [57] following a recent re-organization of the *Microcoleus steenstrupii* complex [35], now known as Coleofasciculaceaea. The reassigned ASVs represented ~2% of all the ASVs in the dataset. We assigned ASVs to the genus or species level when possible. We used a likelihood threshold of 0.7 (pplacer likelihood weight ratio used in iTol [58]) for an ASV to be assigned to a given species or genus; otherwise, it was assigned as “Other Coleofasciculaceae” if placed within the *M. steenstrupii* compex or “not identified” if the likelihood score was below this threshold. We aligned any ASVs that matched multiple taxonomic assignments in Cydrasil to the online Basic Local Alignment Search Tool (BLAST) (https://blast.ncbi.nlm.nih.gov/Blast.cgi) (accessed April 2023) to verify the assignment [59]. We calculated the absolute abundance of cyanobacterial genera or species by multiplying the relative abundance for each ASV by the absolute abundance of the 16S rRNA genes in each sample. 

### 2.5. Statistical Analyses 

We compared cyanobacterial abundances to an initial abundance (calculated as 80% of the control soil community (inoculation control) plus 20% of the community in the reference biocrusts). This calculation is a rough estimate of what we would have measured immediately after inoculation at the cultivation facility and assumes a static community (no growth or decline over the cultivation period of one year). We refer to this community as the “calculated static community”. 

To determine differences in the cyanobacteria communities, we used Non-metric Multidimensional Scaling (NMDS) ordination of Bray–Curtis dissimilarities for ASV relative abundances followed by permutational multivariate analysis of variance (perMANOVA) and PERMDISP using vegan (v 2.6-4) (https://cran.r-project.org/web/packages/vegan/index.html). We then calculated pairwise distances in ordination space for samples of reference biocrusts and cultivated biocrusts with different habitat amelioration treatments (also with vegan in R) and used Kruskal–Wallis tests and Dunn post hoc comparisons to determine how similar the cultivated biocrust cyanobacteria communities were to each of the reference biocrusts, which represent the goal of cultivation. This quantification allowed us to determine how far the cultivated biocrusts had shifted away from the target composition, and for the mixed inoculum, whether the resulting inoculum contained cyanobacteria from one or more reference community. In all cases where we used absolute abundances for the reference biocrust communities, we removed one Colorado Plateau sample that was an outlier for the 16S rRNA gene copies (Grubb’s test, G = 0.395, *p* = 0.002). 

Next, we calculated the absolute abundances for each genus or species and compared their abundances using Kruskal–Wallis tests and pairwise Dunn post hoc comparisons. We calculated alpha diversity (richness) for each cultivated inoculum based on a rarefied ASV table, which we created in phyloseq by removing ASVs with an abundance less than 0.1% and rarefying to 4037 reads per sample (90% of the sample with the lowest read count). We used Kruskal–Wallis tests and Dunn post hoc comparisons to determine the statistical differences in terms of richness. For comparison, we also calculated the richness for each reference biocrust community using a similar approach in which the ASV table was filtered for taxa with an abundance above 0.1% and then rarefied to 90% of the sample with the fewest reads (14,948 reads). We compared the list of unique cyanobacteria genera/species in the reference biocrust to those found in the cultivated biocrusts to understand which taxa were not cultivated and which were detected after cultivation that were not expected in that particular community. 

To understand ASV-level responses to cultivation treatments, we identified cyanobacterial indicator taxa in the reference biocrust microbial communities using the indicspecies package (v1.7.12) [60] with the multipatt function. Our indicator taxa list included ASVs from the Mojave, Sonoran, and taxa shared between the Mojave and Sonoran Deserts. We then compared the absolute abundance of these indicators for the Mojave, Sonoran, and Mixed cultivated inocula using a Kruskal–Wallis non-parametric test with Holm post hoc [61]. For all statistical tests, we assigned a cut-off significance level of 0.05.

Prior to statistical analysis, we assessed cyanobacterial genus/species-level absolute abundances for the two watering regimes using Kruskal–Wallis rank based tests. At the genus/species level, there were no instances where watering regime resulted in different absolute abundances of cyanobacteria (Table S1). Therefore, we included both watering regimes in our analyses without considering it a variable of interest. 

## 3. Results

### 3.1. Cultivated Cyanobacterial Community Composition 

**Figure 2:**
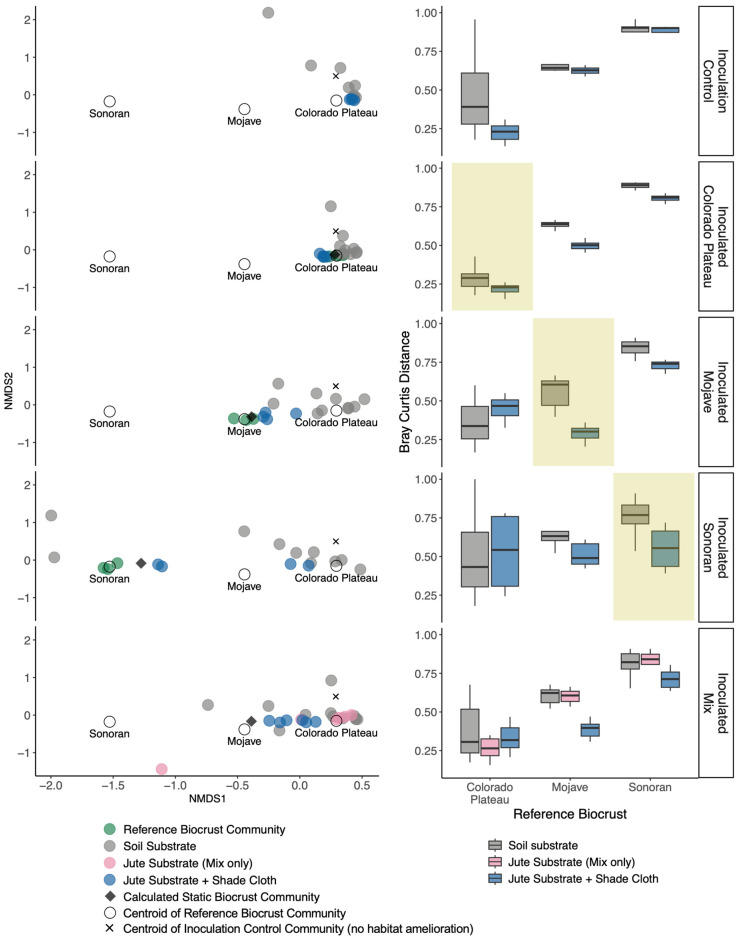
Non-metric Multidimensional Scaling (NMDS) on Bray–Curtis dissimilarities of cyanobacterial relative abundance for inoculation controls and the inoculated cultivated biocrusts (**left panels**). Black diamonds represent the calculated static cyanobacterial community composition (see methods for description). The green points represent the intact cyanobacterial communities at each desert, with the open circles showing the centroid (average) of the intact cyanobacterial community for comparison. The black Xs represent the centroid (average) for inoculation control soils. A single ordination was calculated (stress = 0.129), which is separated into panels for each desert for visual interpretation. Boxplots (**right panels**) indicate the pairwise Bray–Curtis distances from each cultivated inoculum sample to the three reference cyanobacterial communities from the Colorado Plateau, Mojave, and Sonoran deserts. Smaller distance values indicate that the cyanobacterial communities are more similar in ordination space. Yellow highlighted boxes indicate where we would expect more similarity (lower Bray–Curtis distance) in cyanobacterial composition based on the community that was initially inoculated.

Cultivated biocrusts that received inoculum generally did not differ in cyanobacterial community composition from cultivation facility control soils, regardless of the source of the inoculum (e.g., Mojave, Sonoran). This suggests that all of the cultivated biocrusts, in the absence of habitat amelioration, became more like a Colorado Plateau soil through the cultivation process. The ordination plots (Figure 2, left panel) show all of the grey points in similar proximity in ordination space, and the statistical comparisons of the centroids for these points indicated that both their centroids (pairwise perMANOVA, Holm-adjusted *p*-value > 0.05, Supplementary Table S2 and their dispersion (PERMDISP, F = 1.36, *p* = 0.26) are not different. Furthermore, all pairwise Bray–Curtis distances for the inoculated plots without habitat amelioration are shortest to the intact Colorado Plateau biocrust (Figure 2, right panel, Supplementary Table S3). 

Cultivated biocrusts that received inoculum and the jute substrate + shade cloth treatment did not differ in cyanobacterial community composition from the cultivation controls, with habitat amelioration based on pairwise perMANOVA (Supplementary Table S2, Holm-adjusted *p*-value > 0.05). However, dispersion was different (PERMDISP, F = 6.69, *p* = 0.002), meaning that the hot-desert inocula were more variable than the Colorado Plateau inoculum. This suggests that the cyanobacteria communities generally responded similarly to habitat amelioration, with some variability for hot desert biocrusts. The ordination plots (Figure 2, left panel) show the majority of blue points in overlapping regions of the ordination. 

There were also desert-specific ways in which cyanobacteria responded to the habitat amelioration treatment. Both the Colorado Plateau and Mojave inocula were significantly different in ordination space when provided the habitat amelioration treatment (pairwise perMANOVA, Holm-adjusted *p*-value < 0.05, Supplementary Table S2). Moreover, the Mojave cultivated biocrusts shifted toward a Mojave-type biocrust in ordination space and based on the Bray–Curtis distances (Figure 2, right panel). Similarly, in the Sonoran, there was variability in how the cyanobacteria responded to habitat amelioration. In two samples, the community was closer to the Colorado Plateau and in two samples, the community was closer to a Sonoran biocrust. In the mixed inoculum, there is evidence that the cultivated community retained elements of both the Colorado Plateau and Mojave cyanobacterial communities, but only when treated with both jute substrate and shade cloth (pairwise perMANOVA, Holm-adjusted *p*-value < 0.05, Supplementary Table S2). 

**Figure 3:**
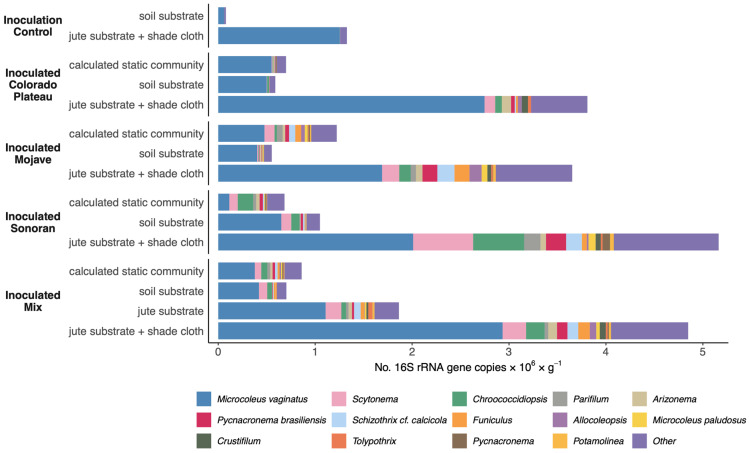
Cyanobacterial absolute abundance after 1 year of cultivation for the control soils, inocula from each desert, and a 1:1:1 inoculum mixture, with and without habitat amelioration (jute substrate and/or shade cloth). The dominant 14 taxa are shown.

**Figure 4:**
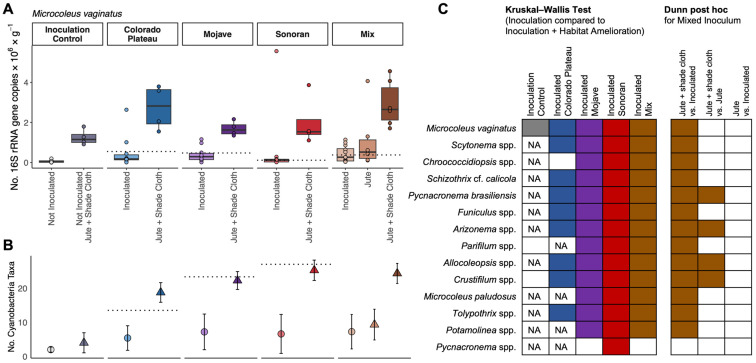
(**A**) Comparison of the absolute abundance of *Microcoleus vaginatus* in each inoculum, including the inoculation control soils, each desert separately, and the mix, with and without habitat amelioration (jute substrate + shade cloth). The boxes represent the lower and upper quartiles, and the solid horizontal line represents the median value. The points represent each replicate. Horizontal dotted lines represent the mean absolute abundance calculated for a static community. (**B**) Average cyanobacteria richness (±standard deviation) for the cultivation controls and the cultivated communities for each treatment at the genus/species level. The horizontal dotted lines indicate the richness of the reference biocrusts from each desert; error bars represent standard deviation. (**C**) Statistical summary of the response of the dominant 14 cyanobacterial genera or species (ordered most to least abundant, top to bottom) to cultivation with versus without habitat amelioration. The colored boxes indicate significant increases in absolute abundance (*p* > 0.05) with habitat amelioration as compared to inoculation only; the white boxes indicate no difference in abundance with habitat amelioration. NA represents cases where the soils being compared had no reads from that taxon. The right set of boxes indicate the pairwise comparisons (Dunn post hoc) of the three habitat amelioration treatments for the mixed inoculum.

The cultivated communities increased in cyanobacterial absolute abundance in response to habitat amelioration (Figure 3). Control soils increased 16-fold in total cyanobacterial 16S rRNA gene copies with habitat amelioration while inoculated soils increased 5-fold to 7-fold. These values approach the number of cyanobacterial 16S rRNA gene copies in reference biocrust communities (Supplementary Figure S3). In most cultivated inocula, *Microcoleus vaginatus* was a dominant taxon, making up anywhere between 40 and 70% of the cyanobacterial community. We expected Sonoran-inoculated soils to contain less abundant *Microcoleus vaginatus* (18%) based on the calculated static community estimate. However, the cultivated community shifted to be more like Colorado Plateau biocrusts during the cultivation process, with 40–60% *M. vaginatus.* For the mixed inoculum with a jute substrate treatment, cyanobacteria increased in abundance (but not as substantially as the jute substrate + shade cloth treatment). Surprisingly, simply providing jute substrate and shade cloth at the cultivation facility (without biocrust inoculation) resulted in a twenty-fold increase in the number of *Microcoleus vaginatus* 16S rRNA gene copies (Figure 4A).

In all cultivated inocula, we expected ~1 × 10^6^ 16S rRNA gene copies for cyanobacteria per gram of soil based on the calculated static community. All of the cultivated biocrusts that only received inoculum were close to this level, indicating that without habitat amelioration treatments, cyanobacteria did not increase above the static community level. Mojave-inoculated biocrusts performed particularly poorly, reaching only 50% of the calculated static community level. This was mostly due to the loss of taxa other than *Microcoleus vaginatus* that were expected to be abundant in a Mojave biocrust but whose abundances were reduced during cultivation. 

In addition to the substantial increase in *Microcoleus vaginatus* in response to habitat amelioration, the majority of dominant cyanobacteria genera or species also responded positively to this treatment. Nine of the fourteen dominant cyanobacteria increased in abundance with jute and shade treatment in all three individual desert inocula and the mixed inocula (Figure 4C, Supplementary Table S4).

We also saw cyanobacterial richness maintained in biocrust inocula grown with habitat amelioration (Figure 4B). The average richness increased three- to four-fold in all inocula when cultivated with jute and shade cloth. There were no significant differences in richness for biocrusts cultivated without jute substrate and shade cloth compared to the inoculation control soils. For biocrusts cultivated with jute substrate and shade cloth, only those inoculated with Mojave and Sonoran biocrusts had cyanobacterial richness that exceeded the controls (Kruskal–Wallis with Dunn post hoc comparison, *p* < 0.05, Supplementary Table S5). Similarly, when comparing the habitat amelioration treatment within the desert inoculum source, all of the biocrusts that received jute substrate and shade cloth had significantly higher cyanobacterial richness than those without habitat amelioration. The mixed inoculum with jute substrate did not differ in richness from the mixed inoculation-only biocrust. Surprisingly, the mixed inocula did not have higher richness at the end of cultivation than any of the individual desert inocula.

For the Colorado Plateau, the mean richness in cultivated biocrust exceeded that present in the reference Colorado Plateau biocrust, which can be explained by the presence of five taxa, *Cyanothece*, *Phormidesmis*, Chroococcales, *Leptolyngbya*, and *Nodosilinea*, which were detected in cultivated samples. The only cyanobacteria genus present in the reference biocrust that did not make it through cultivation was *Stenomitos frigidus*, which comprised 2.6% of reads in the reference community. For cultivated Mojave biocrusts, five genera that were not found in samples of reference Mojave biocrust communities were present after cultivation: *Hormoscilla pringsheimii*, *Nodosilinea*, Chroococcales, *Phormidesmis*, and *Chroococcus* spp. For cultivated Sonoran biocrusts, *Cephalothrix* and *Nodosilinea* were new after cultivation (not present in reference Sonoran biocrusts). Two Sonoran taxa did not make it through cultivation, namely, *Planktothricodies* spp. and *Oculatella* spp., which each made up 1.3% of reads in the reference Sonoran biocrust communities. Genera present in the control soils at the cultivation facility included *M. vaginatus*, *Nostoc*, *Leptolyngbya*, *Parifilum*, *Crustifilum*, and other Undefined Nostocales, Oscillatoriales, and Coleofasciculaceae. 

### 3.2. Indicator Cyanobacteria in Cultivated Mixed Biocrust 

**Figure 5:**
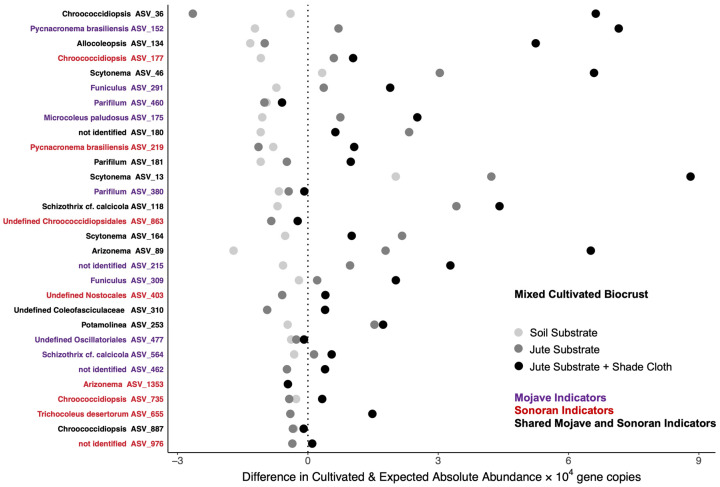
Amplicon sequence variant (ASV)-level mean absolute abundance for indicator taxa in cultivated mixed inoculum compared to calculated static microbial community. Points aligned with the vertical dotted lines indicate that the mean absolute abundance matched that in the calculated community. Points to the right of the vertical dotted line indicate that the cultivated inoculum had a mean absolute abundance higher than expected. Points to the left of the vertical dotted lines indicate a decline in that ASV through the cultivation process. The shading of the points represents plots that were inoculated (light shading), plots that received inoculum and jute substrate (medium shading), or plots that received inoculum and jute substrate + shade cloth (dark shading). Genus or species assignment is shown left of the figure and colored by the desert indicator.

As a way of tracking the abundance of cyanobacterial ASVs unique to each hot desert source, we used indicator taxa analysis, which considers both the presence of ASVs across samples from a particular desert and their uniqueness to that desert. We identified many significant cyanobacterial indicators for both the Mojave and Sonoran deserts, including members of the Coleofasciculaceae family [35] (Figure 5). In a mixed-inoculum cultivation without habitat amelioration, most hot-desert indicator taxa had lower abundance than expected based on the calculated static community. Some ASVs responded well to habitat amelioration, with higher abundance than expected when provided with either a jute substrate or a jute substrate and a shade cloth. Two *Scytonema* ASVs (13 and 46) initially found in both the Mojave and Sonoran deserts but not in the Colorado Plateau biocrusts had higher abundance than expected for all three treatments. Other ASVs responded positively to cultivation with habitat amelioration, including *Chroococcidiopsis* spp., *Pycnacronema* brasiliensis, *Allocoleopsis* spp., and *Arizonema* spp. 

Similar results were observed for the cultivated Mojave and Sonoran cyanobacterial communities. In both cases, habitat amelioration was required for ASV abundance to exceed the calculated static community abundance (Supplementary Figures S4 and S5). 

## 4. Discussion

### 4.1. Tradeoffs in Non-Local Sourcing of Biocrust Inocula

As some species decline in the face of change, there are increasing discussions about the use of assisted migration or genetic modification as tools to support organisms and ecosystem functions on the landscape, and these discussions remain complex (e.g., Hewitt et al., 2011 [62]). The use of including non-local and local biocrust organisms in restoration is a bet-hedging strategy. It is not always possible to predict the climate of a future restoration site while cultivating biocrust inocula, but it is possible that a more diverse biocrust inoculum may provide resistance to a range of abiotic conditions [63], thereby resulting in better biocrust establishment. To create a mixed biocrust community, we moved biocrust communities from the Sonoran and Mojave deserts to the Colorado Plateau. This movement of organisms, or assisted migration, is controversial due to the risk of facilitated invasions. Risks of invasion were reduced in this experiment by using biocrusts from within the same continent [64] and monitoring the growth of invasive plants at the cultivation facility. There are additional potential risks associated with introducing plant seeds and microbial pathogens similar to the application of bioinoculants to enhance plant growth [65,66]. After 1 year of cultivation, some inocula contained both local and hot-adapted microorganisms, but we do not yet know how beneficial this might be under various growth climates. Our cultivation occurred during a very wet and cool year on the Colorado Plateau, which may have influenced the community composition and abundance of the cultivated communities. Further work is needed to determine whether biocrust organisms sourced from other deserts add desirable functional benefits and whether those benefits outweigh the risk of invasion or pathogen spread [67]. Managing the risk of facilitated invasion should be a central part of all future work involving soil- or biocrust-assisted migration. 

With these risks and uncertainties in mind, including particular cyanobacterial taxa in future studies may help limit the organisms introduced, highlight biocrust species/traits that might be particularly successful in recovering systems, and could result in beneficial restoration outcomes. For example, *Scytonema* spp., a thermotolerant heterocystous cyanobacterium that can fix nitrogen, may outcompete other heterocystous cyanobacteria, such as *Tolypthrix* spp. and *Nostoc* spp., in natural systems due to increasing temperatures in drylands [68]. *Scytonema* spp was the second most dominant cyanobacterium in reference biocrust communities and responded positively to cultivation with habitat amelioration. It may be possible to use the field cultivation techniques presented here to include *Scytonema* spp. in restoration. Another cyanobacterium of interest is *Chroococcidiopsis* spp., which had very high abundance in reference Sonoran biocrusts and responded strongly to cultivation with habitat amelioration. Previous work has shown that *Chroococcidiopsis* spp. produces a photo-protective pigment and that it is highly resistant to desiccation and UV exposure [69], and it has also been tested for viability in extreme environments [70]. *Chroococcidiopsis* spp. may be interesting to include in future restoration efforts aiming to include hot-adapted organisms. Particularly for widespread genera such as *Microcoleus* spp. and *Scytonema* spp., future work exploring how variability within and among genera, species, ecotypes, and individuals could help refine and improve innovative restoration options for biocrusts. Genetic approaches such as those used here offer innovative tools among many restoration options.

### 4.2. Shade Cloth Enhances Biocrust Cultivation

We found that cyanobacteria responded strongly to habitat amelioration during cultivation across all three desert sources and even for the control soils. Providing biocrusts with jute substrate and shade cloth massively improved cultivation outcomes, as evidenced by up to an eight-fold increase in *Microcoleus vaginatus* 16S rRNA genes and a three-fold increase in cyanobacterial richness. While the control soils also saw increases in *Microcoleus vaginatus*, inoculation was necessary to cultivate a community with the richness of a reference biocrust, which is desirable for restoration. It is encouraging that cyanobacteria responded strongly to habitat amelioration; they may also specifically respond strongly to shade cloth, as indicated via comparing the jute and jute + shade treatments for the mixed inocula. Shade (e.g., shade cloth) has previously been employed in biocrust cultivation and restoration with promising outcomes [71,72]. The strong effect of shade also points to the potential of using “natural shade” for restoration, such as under the canopy of perennial plants or with downed wood following a fire [73]. 

There may be multiple reasons why shade cloth impacts cyanobacterial survival and establishment in drylands. Shade cloth can act as a physical barrier against wind and water erosion, holding microorganisms and soils in place. Shade cloth can trap moisture at the soil surface, increasing the hydration period during which microbial cells are actively growing, dividing, and repairing. Shade may substantially reduce surface temperatures and UV exposure, especially for microorganisms that do not produce UV-protective pigments. The combined effect is that shade cloth keeps microorganisms in place and reduces a variety of stressors associated with living at the soil surface or barriers to rehabilitation [74]. It is likely that this becomes even more important under hotter and drier conditions or at degraded sites where soils are more unstable and shade-providing plant communities have been disturbed. 

The use of shade in biocrust cultivation is not unique to this study. Shade cloth has been used for cultivating biocrust organisms in greenhouses [75] and fields [71,76,77]. Outdoor cultivation involves accepting more climate variability and uncertainty during cultivation in exchange for being able to produce more inoculum at a scale relevant to land management. Using a habitat amelioration strategy such as those involving shade cloth allows us to mitigate some of the abiotic stress of outdoor cultivation.

### 4.3. Translation to Restoration 

Because of the strong response of biocrust cyanobacteria to cultivation with habitat amelioration, future biocrust restoration efforts should consider employing habitat amelioration strategies throughout both cultivation and application. We found that it is possible to include hot-adapted cyanobacteria in cool-desert biocrust cultivation, but more work is needed to determine the functional outcome of applying a diverse inoculum and to weigh the risks and benefits of biocrust-assisted migration. In particular, assessments of how mixed inocula with local and non-local taxa respond to variable climate conditions and any potential realized benefits in terms of the likelihood of establishment and the long-term survival of biocrust on degraded sites would be exceptionally valuable. Even if there are functional benefits to using a mixed inoculum in restoration, it will be very important to understand the risks involved with biocrust-assisted migration before employing this strategy at scale, and studies such as this can inform others of the benefits and potential unintended consequences, as well as provide insights into restoration options intended for sustaining ecosystems and their functions in the face of accelerating anthropogenic change.

## Figures and Tables

**Table 1:** Biocrust collection site characteristics.

Site Characteristics	Colorado Plateau ^1^	Colorado Plateau ^2^	Mojave Desert ^3^	Sonoran Desert ^4^
Elevation (m)	~1500	~1350	~920	~240
Annual average precipitation (cm)	21.7	22.7	13.9	12.6
Annual average Min–Max Temperature (°C)	6.4–21.8	4.8–21.8	14.8–23.4	15.1–30.6
Soil type ^5^	Aridisol Pocum series	Entisol Thoroughfare series	Aridisol Tonopah series	Aridisol Gunsight series
Biological Soil Crust (Biocrust) Community	Moss- and cyanobacteria-dominant	Moss- and cyanobacteria-dominant	Lichen- and cyanobacteria-dominant	Lichen- and cyanobacteria-dominant
Ecological Site ^6^	Semidesert Shallow Sandy Loam (Blackbrush)	Desert Sandy Loam (Fourwing Saltbush)	Arid Active Alluvial Fans	Limy Upland and Desert Pavements

^1^ Climate data from Western Regional Climate Center (WRCC) (wrcc.dri.edu) (accessed February 2023), Arches NP HQS Station. ^2^ Climate data from WRCC, Moab Station. ^3^ Climate data from WRCC, Jean Station. ^4^ Climate data from WRCC, Gila Bend 3 ENE Station. ^5^ Soil data from NRCS SoilWeb (casoilresource.lawr.ucdavis.edu) (accessed February 2023). ^6^ Ecological Site descriptions from the Ecosystem Dynamics Interpretive Tool (edit.jornada.nmsu.edu) (accessed February 2023).

## Data Availability

The data presented in this study are openly available at the Sequence Read Archive (SRA) through the National Center for Biotechnology Information (NCBI) under BioProject PRJNA1025292. Accession numbers associated with each sample used in this work are provided in Table S8 and sample descriptions can be found in the metadata table for this BioProject.

## Supplementary Materials

The following supporting information can be downloaded at: https://www.mdpi.com/article/10.3390/microorganisms11102570/s1, Table S1: Statistical comparison of cultivated cyanobacteria due to watering regime; Table S2: Statistical output for cultivated cyanobacterial ordination; Table S3: Statistical output for reference biocrust cyanobacterial communities, Table S4: Statistical output for cultivated biocrust cyanobacterial communities; Table S5: Statistical output for cultivated cyanobacterial richness; Table S6: Statistical output of microbial phyla of reference biocrust communities; Table S7: Statistical output of cyanobacterial genera or species of reference biocrust communities; Table S8: Sample accessions for NCBI SRA; Figure S1: Biocrust cultivation climate; Figure S2: Biocrust cultivation materials; Figure S3: Microbial composition of reference biocrust communities; Figure S4: Cyanobacterial indicator taxa found in cultivated Mojave-inoculated biocrust, Figure S5: Cyanobacterial indicator taxa found in cultivated Sonoran-inoculated biocrust; Figure S6: Relationship between cyanobacteria and mean annual temperature.
