# Supplementary material for: Cultivating Resilience in Dryland Soils: An Assisted Migration Approach to Biological Soil Crust Restoration
Source: Microorganisms. 2023 Oct 15;11(10):2570. doi: 10.3390/microorganisms11102570 (PMC10608944; doi:10.3390/microorganisms11102570)
Supplement: Supplementary file 1 [file microorganisms-11-02570-s001.zip › microorganisms-2561667-supplementary.pdf]

microorganisms

MDPI

Supplemental Information

Supplemental Table S1. Kruskal-Wallis test of cyanobacterial genus or species-level differences in absolute abundances for cultivated biocrust communities with two different watering regimes (df = 1). Biocrusts were cultivated with two different watering regimes: A) 1.5 hours per day, three times per week in spring and fall or B) 3 hours per day, once per week in spring and fall. Based on these results, we excluded watering regime from the main study analysis. Significant differences in watering regime are bolded.

	**Kruskal-Wallis**		
	Inoculum Source (Desert)	Chi-squared	P
Microcoleus vaginatus	Inoculation Control	2.08	0.1489
	Colorado Plateau	0.18	0.6698
	Mojave	2.91	0.08808
	Sonoran	0.18	0.6698
	Mix	0	1
Scytonema spp.	Inoculation Control	NA	
	Colorado Plateau	3.25	0.07163
	Mojave	0.47	0.4951
	Sonoran	0.05	0.8291
	Mix	0.11	0.7398
Chroococcidiopsis spp.	Inoculation Control	NA	
	Colorado Plateau	1.74	0.1869
	Mojave	0.41	0.5212
	Sonoran	0.18	0.6698
	Mix	0.65	0.4201
Schizothrix cf-calicola	Inoculation Control	NA	
	Colorado Plateau	NA	
	Mojave	0.07	0.7931
	Sonoran	0.21	0.6481
	Mix	0.80	0.372
Pycnacronema brasiliensis	Inoculation Control	NA	
	Colorado Plateau	3.33	0.06789
	Mojave	0.62	0.4313
	Sonoran	0.21	0.6492
	Mix	0.15	0.7032
Funiculus spp.	Inoculation Control	NA	
	Colorado Plateau	1.5	0.2207
	Mojave	0.23	0.631
	Sonoran	1.10	0.2941
	Mix	0.74	0.3912
Arizonema spp.	Inoculation Control	NA	
	Colorado Plateau	NA	
	Mojave	0.02	0.8791
	Sonoran	0.21	0.6481
	Mix	1	0.3173
Parifilum spp.	Inoculation Control	NA	
	Colorado Plateau	NA	
	Mojave	1.48	0.2235
	Sonoran	0	1
	Mix	0.02	0.902
Allocoleopsis spp.	Inoculation Control	NA	
	Colorado Plateau	1.5	0.2207
	Mojave	0.07	0.7931
	Sonoran	0.21	0.6481
	Mix	1	0.3173
Crustifilum spp.	Inoculation Control	NA	
	Colorado Plateau	NA	

	Mojave	NA	
	Sonoran	1.5	0.2207
	Mix	NA	
Microcoleus paludosus	Inoculation Control	NA	
	Colorado Plateau	NA	
	Mojave	2.48	0.1155
	Sonoran	1.5	0.2207
	Mix	1.53	0.2155
Tolypothrix spp.	Inoculation Control	NA	
	Colorado Plateau	1.72	0.1897
	Mojave	1.5	0.2207
	Sonoran	NA	
	Mix	0.02	0.902
Potamolinea spp.	Inoculation Control	NA	
	Colorado Plateau	NA	
	Mojave	1.48	0.2235
	Sonoran	0.21	0.6481
	Mix	1	0.3173
Pycnacronema spp.	Inoculation Control	NA	
	Colorado Plateau	NA	
	Mojave	0.67	0.4142
	Sonoran	NA	
	Mix	1	0.3173

Supplemental Table S2. perMANOVA, pairwise perMANOVA, and PERMDISP results comparing Bray-Curtis dissimilarities on cyanobacterial community relative abundances for each inoculum source. A) Determining the effect of inoculation by comparing inoculated soils to control soils. In this case, we are comparing the Inoculation Control samples in the ordination to each of the subsequent inoculated samples in the ordination separately for without and with habitat amelioration. B) Determining the effect of habitat amelioration by comparing no habitat amelioration to the jute (mixed inoculum only) or jute + shade cloth treatments. In this case, we are comparing the grey and blue points on the ordination within inoculum source. Only relevant pairwise perMANOVA results are included. PERMDISP tests for differences in the dispersion of community composition between groups and can help determine whether the significant perMANOVA is due to differences in centroids or due to differences in dispersion (variability between samples). Significant tests are bolded.

Comparison to Controls

	**perMANOVA**				**Pairwise perMANOVA**		**PERMDISP**	
	Pseudo-F	df	R^2	p	Comparison	Holm adjusted p	F	p
Inoculation	**1.8752**	**4**	**0.1428**	**0.003**	Soil – CP Inoc	0.26	1.3633	0.2617
					Soil – MJ Inoc	0.43		
					Soil – SN Inoc	0.34		
					Soil – Mix Inoc	0.43		
					Mix Inoc – CP Inoc	0.43		
					Mix Inoc – MJ Inoc	0.52		
					Mix Inoc – SN Inoc	0.43		
Inoculation + Habitat Amelioration	**6.7218**	**4**	**0.6126**	**0.001**	Soil – CP Inoc	0.17	**6.6919**	**0.002**
					Soil – MJ Inoc	0.17		
					Soil – SN Inoc	0.17		
					Soil – Mix Inoc	0.05		
					Mix Inoc – CP Inoc	0.13		
					Mix Inoc – MJ Inoc	0.17		
					Mix Inoc – SN Inoc	0.17		

Cultivated Inoculum, Comparison for Habitat Amelioration

	**perMANOVA**				**Pairwise perMANOVA**		**PERMDISP**	
	Pseudo-F	df	R^2	p	Comparison	Holm adjusted p	F	p
Inoculation Control	1.6378	1	0.1407	0.104	NA		4.6784	0.0558
Colorado Plateau	**2.5147**	**1**	**0.1733**	**0.033**	NA		2.4871	0.1408
Mojave	**4.9282**	**1**	**0.2911**	**0.002**	NA		4.4229	0.0572
Sonoran	1.5372	1	0.1134	0.138	NA		0.8784	0.3671
Mix	**1.9838**	**2**	**01589**	**0.032**	Inoc v. Jute	0.38	0.8765	0.4309
					**Inoc v. Amelior**	**0.03**		
					Jute v. Amelior	0.14		

Soil = inoculation control soils, CP = Colorado Plateau inoculated soils, MJ = Mojave inoculated soils, SN = Sonoran inoculated soils, MX = 1:1:1 mix of CP, MJ, and SN inoculated soils. Note, there was no control jute only treatment, so we did not assess differences for the mixed inoculation + jute substrate. Inoc = cultivated biocrusts that received only inoculum, Amelior = cultivated biocrusts that received inoculum and jute substrate + shade cloth, Jute = cultivated biocrusts that received inoculum and jute substrate

Supplemental Table S3. Kruskal-Wallis rank based comparison of pairwise Bray-Curtis distances of cultivated biocrust cyanobacteria communities compared to reference biocrust cyanobacteria communities as displayed in Fig. 2 of the main text. We include only the relevant comparisons based on which type of source biocrust community was used to cultivate the inoculum. We used Dunn test post-hoc pairwise comparisons with holm p-value adjustments. Significant tests are bold.

	**Kruskal-Wallis**			**Dunn post-hoc**		
	Chi-squared	df	P	Comparison	Z	Holm adjusted p
Inoculation Control	**95.594**	**5**	**<2.2e-16**	**CP-Soil v. CP-Amelior**	**-3.344**	**0.0066**
				MJ-Soil v. MJ-Amelior	-1.141	0.5074
				SN-Soil v. SN-Amelior	-0.571	0.5682
				**CP-Soil v. MJ-Soil**	**-3.516**	**0.0040**
				MJ-Soil v. SN-Soil	-2.464	0.0825
				**CP-Soil v. SN-Soil**	**-6.361**	**2.802e-9**
				**CP-Amelior v. MJ-Amelior**	**-3.709**	**0.0021**
				MJ-Amelior v. SN-Amelior	-2.237	0.1266
				**CP-Amelior v. SN-Amelior**	**-6.292**	**4.068e-9**
Colorado Plateau Inoculated	**107.62**	**5**	**<2.2e-16**	CP-Inoc v. CP-Amelior	-2.247	0.1232
				MJ- Inoc v. MJ-Amelior	-1.215	0.6732
				SN- Inoc v. SN-Amelior	-1.157	0.2472
				**CP- Inoc v. MJ- Inoc**	**-4.243**	**2.211e-4**
				MJ- Inoc v. SN- Inoc	-2.587	0.0677
				**CP- Inoc v. SN- Inoc**	**-7.231**	**6.708e-12**
				**CP-Amelior v. MJ-Amelior**	**-3.859**	**0.0010**
				**MJ-Amelior v. SN-Amelior**	**-2.165**	**1.214e-6**
				**CP-Amelior v. SN-Amelior**	**-6.359**	**2.635e-9**
Mojave Inoculated	**128.37**	**5**	**<2.2e-16**	CP- Inoc v. CP-Amelior	2.386	0.0853
				**MJ- Inoc v. MJ-Amelior**	**-4.469**	**7.071e-5**
				SN- Inoc v. SN-Amelior	-1.187	0.2353
				**CP- Inoc v. MJ- Inoc**	**-4.979**	**6.405e-6**
				**MJ- Inoc v. SN- Inoc**	**-4.124**	**2.973e-4**
				**CP- Inoc v. SN- Inoc**	**-9.741**	**3.020e-21**
				CP-Amelior v. MJ-Amelior	2.798	0.0308
				**MJ-Amelior v. SN-Amelior**	**-5.355**	**9.425e-7**
				**CP-Amelior v. SN-Amelior**	**-3.385**	**0.0050**
Sonoran Inoculated	**37.315**	**5**	**5.179e-7**	CP- Inoc v. CP-Amelior	0.695	1
				MJ- Inoc v. MJ-Amelior	-2.180	0.2927
				**SN- Inoc v. SN-Amelior**	**-3.052**	**0.0250**
				**CP- Inoc v. MJ- Inoc**	**-3.100**	**0.0232**
				MJ- Inoc v. SN- Inoc	-2.102	0.3199
				**CP- Inoc v. SN- Inoc**	**-5.527**	**4.881e-7**
				CP-Amelior v. MJ-Amelior	0.620	1
				MJ-Amelior v. SN-Amelior	-0.600	1
				CP-Amelior v. SN-Amelior	-0.073	0.9421
Mix Inoculated	**183.92**	**8**	**<2.2e-16**	CP- Inoc v. CP-Jute	-0.790	1
				MJ- Inoc v. MJ-Jute	-0.279	1
				SN- Inoc v. SN-Jute	0.091	0.9277
				CP- Inoc v. CP-Amelior	-0.445	1
				MJ- Inoc v. MJ-Amelior	-2.626	0.1211
				SN- Inoc v. SN-Amelior	-1.456	1
				CP- Jute v. CP-Amelior	0.299	1
				MJ- Jute v. MJ-Amelior	-2.032	0.5477
				SN- Jute v. SN-Amelior	-1.340	1
				**CP- Inoc v. MJ- Inoc**	**-4.721**	**5.643e-5**
				**MJ- Inoc v. SN- Inoc**	**-4.084**	**9.742e-4**
				**CP- Inoc v. SN- Inoc**	**-9.436**	**1.390e-19**
				**CP-Jute v. MJ-Jute**	**-3.618**	**0.0060**
				**MJ-Jute v. SN-Jute**	**-3.208**	**0.0201**
				**CP-Jute v. SN-Jute**	**-7.322**	**7.828e-12**

CP-Amelior v. MJ-Amelior	-1.027	1
**MJ-Amelior v. SN-Amelior**	**-3.900**	**0.0020**
**CP-Amelior v. SN-Amelior**	**-5.531**	**8.600e-7**

CP = reference biocrust Colorado Plateau, MJ = reference biocrust Mojave, SN = reference biocrust Sonoran, dashes indicate distance between the reference biocrust community and the cultivated biocrust community treatment (soil, inoc, or amelior), Soil = inoculation control soils, Inoc = inoculated without habitat amelioration treatments, Amelior = jute substrate and shade cloth, Jute = jute substrate.

Supplemental Table S4. Kruskal-Wallis test and Dunn post-hoc pairwise comparison of genus-level or species-level differences in absolute abundances for cultivated biocrust communities with and without habitat amelioration. Significant tests are bolded.

	**Kruskal-Wallis**			**Dunn post-hoc (Mixed Inocula only)**		
	Inoculum Source (Desert)	Chi-squared	p	Comparison	Z	Holm adjusted p
Microcoleus vaginatus	**Inoculation Control**	**7.3846**	**0.0066**			
	**Colorado Plateau**	**6.48**	**0.0109**			
	**Mojave**	**8**	**0.0047**			
	**Sonoran**	**5.12**	**0.0237**			
	**Mix**	**12.045**	**0.0024**	**Jute + shade cloth vs. Inoculated**	**3.46**	**0.0016**
				Jute + shade cloth vs. Jute	2.16	0.061
				Jute vs. Inoculated	0.97	0.3339
Scytonema spp.	Inoculation Control	NA	NA			
	**Colorado Plateau**	**7.02**	**0.0081**			
	**Mojave**	**8.368**	**0.0038**			
	**Sonoran**	**5.165**	**0.0230**			
	**Mix**	**6.293**	**0.043**	**Jute + shade cloth vs. Inoculated**	**2.43**	**0.0448**
				Jute + shade cloth vs. Jute	1.90	0.1145
				Jute vs. Inoculated	0.24	0.8114
Chroococcidiopsis spp.	Inoculation Control	NA	NA			
	Colorado Plateau	2.9447	0.0862			
	**Mojave**	**5.12**	**0.0236**			
	**Sonoran**	**7.2359**	**0.0071**			
	**Mix**	**8.9138**	**0.0116**	**Jute + shade cloth vs. Inoculated**	**2.95**	**0.0096**
				Jute + shade cloth vs. Jute	2.08	0.0743
				Jute vs. Inoculated	0.54	0.5874
Schizothrix cf-calicola	Inoculation Control	NA	NA			
	**Colorado Plateau**	**8.7128**	**0.0032**			
	**Mojave**	**9.1228**	**0.0025**			
	**Sonoran**	**7.0887**	**0.0078**			
	**Mix**	**10.306**	**0.0058**	**Jute + shade cloth vs. Inoculated**	**3.17**	**0.0045**
				Jute + shade cloth vs. Jute	2.23	0.0512
				Jute vs. Inoculated	0.60	0.5519
Pycnacronema brasiliensis	Inoculation Control	NA	NA			
	**Colorado Plateau**	**9.8113**	**0.0017**			
	**Mojave**	**9.1228**	**0.0025**			
	**Sonoran**	**5.3554**	**0.0207**			
	**Mix**	**14.455**	**0.0007**	**Jute + shade cloth vs. Inoculated**	**3.79**	**0.0004**
				**Jute + shade cloth vs. Jute**	**2.42**	**0.031**
				Jute vs. Inoculated	1.00	0.316
Funiculus spp.	Inoculation Control	NA	NA			
	**Colorado Plateau**	**6.598**	**0.0102**			
	**Mojave**	**8.667**	**0.0032**			
	**Sonoran**	**9.122**	**0.0025**			
	**Mix**	**11.49**	**0.0032**	**Jute + shade cloth vs. Inoculated**	**3.37**	**0.002**
				Jute + shade cloth vs. Jute	2.23	0.051
				Jute vs. Inoculated	0.79	0.428
Arizonema spp.	Inoculation Control	NA	NA			
	**Colorado Plateau**	**12.552**	**0.0004**			
	**Mojave**	**9.8113**	**0.0017**			
	**Sonoran**	**7.9472**	**0.0048**			
	**Mix**	**14.399**	**0.0007**	**Jute + shade cloth vs. Inoculated**	**3.79**	**0.0004**
				**Jute + shade cloth vs. Jute**	**2.25**	**0.0485**
				Jute vs. Inoculated	1.19	0.2331
Parifilum spp.	Inoculation Control	2	0.1573			
	Colorado Plateau	NA	NA			

	**Mojave**	**8.8547**	**0.0029**			
	**Sonoran**	**7.390**	**0.0066**			
	**Mix**	**10.654**	**0.0049**	**Jute + shade cloth vs. Inoculated**	**3.26**	**0.0033**
				Jute + shade cloth vs. Jute	1.82	0.1365
				Jute vs. Inoculated	1.16	0.2469
Allocoleopsis spp.	Inoculation Control	NA	NA			
	**Colorado Plateau**	**10.866**	**0.00098**			
	**Mojave**	**9.1228**	**0.00252**			
	**Sonoran**	**3.9116**	**0.048**			
	**Mix**	**18.487**	**9.67e-04**	**Jute + shade cloth vs. Inoculated**	**4.14**	**0.0001**
				**Jute + shade cloth vs. Jute**	**3.33**	**0.0017**
				Jute vs. Inoculated	0.30	0.768
Crustifilum spp.	Inoculation Control	2	0.1573			
	**Colorado Plateau**	**12.552**	**0.0004**			
	**Mojave**	**12.552**	**0.0004**			
	**Sonoran**	**9.8063**	**0.0017**			
	**Mix**	**17.03**	**0.0002**	**Jute + shade cloth vs. Inoculated**	**4.16**	**9.69e-5**
				**Jute + shade cloth vs. Jute**	**2.53**	**2.28e-2**
				Jute vs. Inoculated	1.23	2.17e-1
Microcoleus paludosus	Inoculation Control	NA	NA			
	Colorado Plateau	NA	NA			
	**Mojave**	**5.8386**	**0.0157**			
	**Sonoran**	**8.8012**	**0.0030**			
	**Mix**	**8.1825**	**0.0167**	**Jute + shade cloth vs. Inoculated**	**2.85**	**0.013**
				Jute + shade cloth vs. Jute	1.88	0.121
				Jute vs. Inoculated	0.68	0.496
Tolypothrix spp.	Inoculation Control	NA	NA			
	**Colorado Plateau**	**9.1228**	**0.0025**			
	**Mojave**	**6.5975**	**0.0102**			
	**Sonoran**	**12.552**	**0.0004**			
	**Mix**	**6.8207**	**0.0330**	**Jute + shade cloth vs. Inoculated**	**2.60**	**0.028**
				Jute + shade cloth vs. Jute	1.69	0.182
				Jute vs. Inoculated	0.65	0.515
Potamolinea spp.	Inoculation Control	NA	NA			
	Colorado Plateau	NA	NA			
	**Mojave**	**9.811**	**0.0017**			
	**Sonoran**	**7.089**	**0.0078**			
	**Mix**	**7.277**	**0.0263**	**Jute + shade cloth vs. Inoculated**	**2.68**	**0.022**
				Jute + shade cloth vs. Jute	1.80	0.144
				Jute vs. Inoculated	0.60	0.546
Pycnacronema spp.	Inoculation Control	NA	NA			
	Colorado Plateau	NA	NA			
	Mojave	0.3365	0.5618			
	**Sonoran**	**12.552**	**0.0004**			
	Mix	4.732	0.0939	NA		

* CP = Colorado Plateau, MJ = Mojave, SN = Sonoran

Supplemental Table S5. Kruskal-Wallis test and Dunn post-hoc pairwise comparison of cyanobacterial richness for cultivated biocrust communities. A) Determining the effect of inoculation by comparing inoculated soils to control soils. B) Determining the effect of habitat amelioration by comparing no habitat amelioration to the jute (mixed inoculum only) or jute + shade cloth treatments. Only relevant pairwise results are included. Significant tests are bolded.

Comparison to Cultivation Controls

	**Kruskal-Wallis**			**Dunn post-hoc**		
	Chi-squared	df	p	Comparison	Z	Holm adjusted p
Inoculation	**12.458**	**4**	**0.0143**	Soil – CP Inoc	-2.540	0.0777
				**Soil – MJ Inoc**	**-3.100**	**0.0194**
				Soil – SN Inoc	-2.641	0.0660
				**Soil – MX Inoc**	**-3.069**	**0.0193**
				Mix Inoc – CP Inoc	-0.458	1
				Mix Inoc – MJ Inoc	0.1626	1
				Mix Inoc – SN Inoc	0.3453	1
Inoculation + Habitat Amelioration	**15.555**	**4**	**0.0037**	Soil – CP Inoc	-1.0942	1
				Soil – MJ Inoc	-2.0516	0.2814
				**Soil – SN Inoc**	**-3.2826**	**0.0103**
				**Soil – MX Inoc**	**-3.2163**	**0.0117**
				Mix Inoc – CP Inoc	-2.0177	0.2617
				Mix Inoc – MJ Inoc	-0.9689	0.9978
				Mix Inoc – SN Inoc	-0.3796	0.7043

Cultivated Inoculum, Comparison for Habitat Amelioration

	**Kruskal-Wallis**			**Dunn post-hoc**		
	Chi-squared	df	p	Comparison	Z	Holm adjusted p
Control	0.790	1	0.374	NA		
Colorado Plateau	**7.741**	**1**	**0.0054**			
Mojave	**8.071**	**1**	**0.0045**			
Sonoran	**8.161**	**1**	**0.0043**			
Mix	**13.487**	**2**	**0.0012**	Inoc v. Inoc + Substrate	0.674	0.5005
				**Inoc v. Inoc + Amelior**	**3.628**	**0.0009**
				**Inoc + Substrate v. Inoc + Amelior**	**2.559**	**0.0210**

* Soil = inoculation controls soils, CP = Colorado Plateau inoculated soils, MJ = Mojave inoculated soils, SN = Sonoran inoculated soils, MX = 1:1:1 mix of CP, MJ, and SN inoculated soils, Inoc = cultivated biocrusts that received only inoculum, Amelior = cultivated biocrusts that received inoculum and substrate + cover

Supplemental Table S6. Kruskal-Wallis test and Dunn post-hoc pairwise comparison of phylum-level differences in absolute abundances (number of 16S rRNA gene copies) for reference biocrust communities from the three deserts. Significant tests are bolded.

	**Kruskal-Wallis**			**Dunn post-hoc**		
	Chi-squared	df	P	Comparison	Z	Holm adjusted p
Cyanobacteria	4.7758	2	0.09182		NA	
Bacteroidota	**7.0788**	**2**	**0.02903**	CP – MJ	1.707	0.1758
				**CP – SN**	**2.532**	**0.0340**
				MJ - SN	0.739	0.4602
Chloroflexi	2.1697	2	0.338		NA	
Acidobacteriota	4.1697	2	0.1243		NA	
Proteobacteria	0.7758	2	0.6785		NA	
Actinobacteriota	4.2424	2	0.1199		NA	
Crenarchaeota	0.8485	2	0.6543		NA	
Verrucomicrobiota	5.2606	2	0.0721		NA	
Planctomycetota	3.3818	2	0.1844		NA	

* CP = Colorado Plateau, MJ = Mojave, SN = Sonoran

Supplemental Table S7. Kruskal-Wallis test and Dunn post-hoc pairwise comparison of genus-level or species-level differences in absolute abundances for reference biocrust communities from the three deserts. Significant tests are bolded.

	Kruskal-Wallis			Dunn post-hoc		
	Chi-squared	df	P	Comparison	Z	Holm adjusted p
Microcoleus vaginatus	**6.2308**	**2**	**0.04714**	CP – MJ	-0.330	0.7412
				CP – SN	2.146	0.0636
				MJ - SN	2.216	0.0802
Scytonema	**7.6712**	**2**	**0.02159**	**CP – MJ**	**-2.483**	**0.0391**
				CP – SN	-2.069	0.0771
				MJ - SN	0.370	0.7113
Chroococcidiopsis	**8.0485**	**2**	**0.01788**	CP – MJ	-1.431	0.3047
				**CP – SN**	**-2.807**	**0.0150**
				MJ - SN	-1.231	0.2184
Parifilum	**9.0667**	**2**	**0.01074**	**CP – MJ**	**-2.887**	**0.0117**
				CP – SN	-1.876	0.1212
				MJ - SN	0.904	0.3662
Arizonema	**6.4049**	**2**	**0.04066**	CP – MJ	-1.490	0.2726
				**CP – SN**	**-2.455**	**0.0422**
				MJ - SN	-0.864	0.3878
Pycnacronema brasiliensis	**7.5495**	**2**	**0.02294**	CP – MJ	-2.345	0.0571
				CP – SN	-2.207	0.0546
				MJ - SN	0.123	0.9018
Schizothrix cf-calcicola	**8.7273**	**2**	**0.01273**	**CP – MJ**	**-2.890**	**0.0116**
				CP – SN	-1.651	0.1973
				MJ - SN	1.108	0.2679
Funiculus	**7.1495**	**2**	**0.02802**	**CP – MJ**	**-2.521**	**0.0351**
				CP – SN	0.120	0.9044
				MJ - SN	2.148	0.0634
Allocoleopsis	**7.1111**	**2**	**0.02857**	**CP – MJ**	**-2.667**	**0.0230**
				CP – SN	-1.000	0.3173
				MJ - SN	1.491	0.2721
Microcoleus paludosus	**9.6**	**2**	**0.00823**	**CP – MJ**	**-3.031**	**0.0073**
				CP – SN	-1.732	0.1665
				MJ - SN	1.162	0.2453
Crustifilum	1.5099	2	0.47	CP – MJ	-0.055	0.9560
				CP – SN	-1.159	0.7398
				MJ - SN	-0.987	0.6473
Tolypothrix	5.3455	2	0.06906	CP – MJ	-2.229	0.0774
				CP – SN	-1.404	0.3208
				MJ - SN	0.739	0.4602
Pycnacronema	5.7724	2	0.05579	CP – MJ	-1.116	0.2645
				CP – SN	-2.391	0.0504
				MJ - SN	-1.141	0.5081
Potamolinea	**9.6**	**2**	**0.00823**	**CP – MJ**	**-3.031**	**0.0073**
				CP – SN	-1.732	0.1665
				MJ - SN	1.162	0.2453

* CP = Colorado Plateau, MJ = Mojave, SN = Sonoran

Supplemental Table S8. Sample accessions for publicly available sequencing data associated with this project on NCBI Sequence Read Archive. Descriptions of each sample can be found in the associated metadata table on NCBI under BioProject PRJNA1025292.

Sample	Accession	Sample	Accession	Sample	Accession
cesu1MJinitialaDNA	SAMN37723039	cesu139MB2MJ2BN353	SAMN37722973	cesu187MB2MX2BJC262	SAMN37723026
cesu3MJinitialcDNA	SAMN37723182	cesu140MB2MJ2BN354	SAMN37722975	cesu188MB2MX2BJC263	SAMN37723027
cesu2MJinitialbDNA	SAMN37723145	cesu141MB2CP2BN1	SAMN37722976	cesu189MB2MX2BJC404	SAMN37723028
cesu4SNinitialaDNA	SAMN37723193	cesu142MB2CP2BN2	SAMN37722977	cesu190MB2MX2BJC405	SAMN37723029
cesu5SNinitialbDNA	SAMN37723204	cesu143MB2CP2BN368	SAMN37722978	cesu191Blank32DNA820	SAMN37723030
cesu6SNinitialcDNA	SAMN37723215	cesu144MB2CP2BN369	SAMN37722979	cesu192Blank28DNA820	SAMN37723031
cesu7CPinitialaDNA	SAMN37723226	cesu145MB2CP2BN370	SAMN37722980	cesu115MB2CP2AJC86204	SAMN37722951
cesu8CPinitialbDNA	SAMN37723237	cesu146MB2CP2BN371	SAMN37722981	cesu116MB2CP2AJC86205	SAMN37722953
cesu9CPinitialc	SAMN37723248	cesu147MB2SN2BN244	SAMN37722982	cesu117MB2CP2AJC71314	SAMN37722955
cesu307CPUSUinitiala	SAMN37723153	cesu148MB2SN2BN245	SAMN37722983	cesu118MB2CP2AJC71315	SAMN37722957
cesu308CPUSUinitialb	SAMN37723154	cesu149MB2SN2BN246	SAMN37722984	cesu111MB2MJ2AJC8741	SAMN37722944
cesu309CPUSUinitialc	SAMN37723155	cesu150MB2SN2BN247	SAMN37722986	cesu112MB2MJ2AJC8744	SAMN37722946
cesu99MB2CP2AN86201	SAMN37723247	cesu151MB2SN2BN384	SAMN37722987	cesu113MB2MJ2AJC70329	SAMN37722948
cesu100MB2CP2AN86203	SAMN37722931	cesu152Blank30DNA820	SAMN37722988	cesu114MB2MJ2AJC70330	SAMN37722950
cesu101MB2CP2AN71308	SAMN37722932	cesu153MB2SN2BN386	SAMN37722989	cesu115MB2SN2AJC85237	SAMN37722952
cesu102MB2CP2AN71310	SAMN37722933	cesu154MB2MX2BN257	SAMN37722990	cesu116MB2SN2AJC85239	SAMN37722954
cesu103MB2MJ2AN8739	SAMN37722934	cesu155MB2MX2BN258	SAMN37722991	cesu117MB2SN2AJC72297	SAMN37722956
cesu104MB2MJ2AN8740	SAMN37722935	cesu156MB2MX2BN259	SAMN37722992	cesu118MB2SN2AJC72299	SAMN37722958
cesu105MB2MJ2AN70324	SAMN37722936	cesu157MB2MX2BN401	SAMN37722993	cesu127MB2C2AJC53424	SAMN37722960

cesu106MB2MJ2AN70327	SAMN37722937	cesu158MB2MX2BN402	SAMN37722994	cesu128MB2C2AJC53425	SAMN37722961
cesu107MB2SN2AN85232	SAMN37722938	cesu159MB2MX2BN403	SAMN37722995	cesu129MB2C2AJC53426	SAMN37722962
cesu108MB2SN2AN85234	SAMN37722939	cesu160MB2MX2AN221	SAMN37722997	cesu130MB2C2AJC53427	SAMN37722964
cesu109MB2SN2AN72293	SAMN37722940	cesu161MB2MX2AN222	SAMN37722998		
cesu110MB2SN2AN72295	SAMN37722942	cesu162MB2MX2AN224	SAMN37722999		
cesu111MB2C2AN53420	SAMN37722943	cesu163MB2MX2AN340	SAMN37723000		
cesu112MB2C2AN53421	SAMN37722945	cesu164MB2MX2AN342	SAMN37723001		
cesu113MB2C2AN53422	SAMN37722947	cesu165MB2MX2AN343	SAMN37723002		
cesu114MB2C2AN53423	SAMN37722949	cesu178Blank31DNA820	SAMN37723016		
cesu131MB2C2BN276	SAMN37722965	cesu179MB2MX2BJN264	SAMN37723017		
cesu132MB2C2BN277	SAMN37722966	cesu180MB2MX2BJN266	SAMN37723019		
cesu133MB2C2BN278	SAMN37722967	cesu181MB2MX2BJN267	SAMN37723020		
cesu134MB2C2BN279	SAMN37722968	cesu182MB2MX2BJN408	SAMN37723021		
cesu135MB2MJ2BN17	SAMN37722969	cesu183MB2MX2BJN409	SAMN37723022		
cesu136MB2MJ2BN18	SAMN37722970	cesu184MB2MX2BJN410	SAMN37723023		
cesu137MB2MJ2BN19	SAMN37722971	cesu185MB2MX2BJC260	SAMN37723024		
cesu138MB2MJ2BN352	SAMN37722972	cesu186MB2MX2BJC261	SAMN37723025		

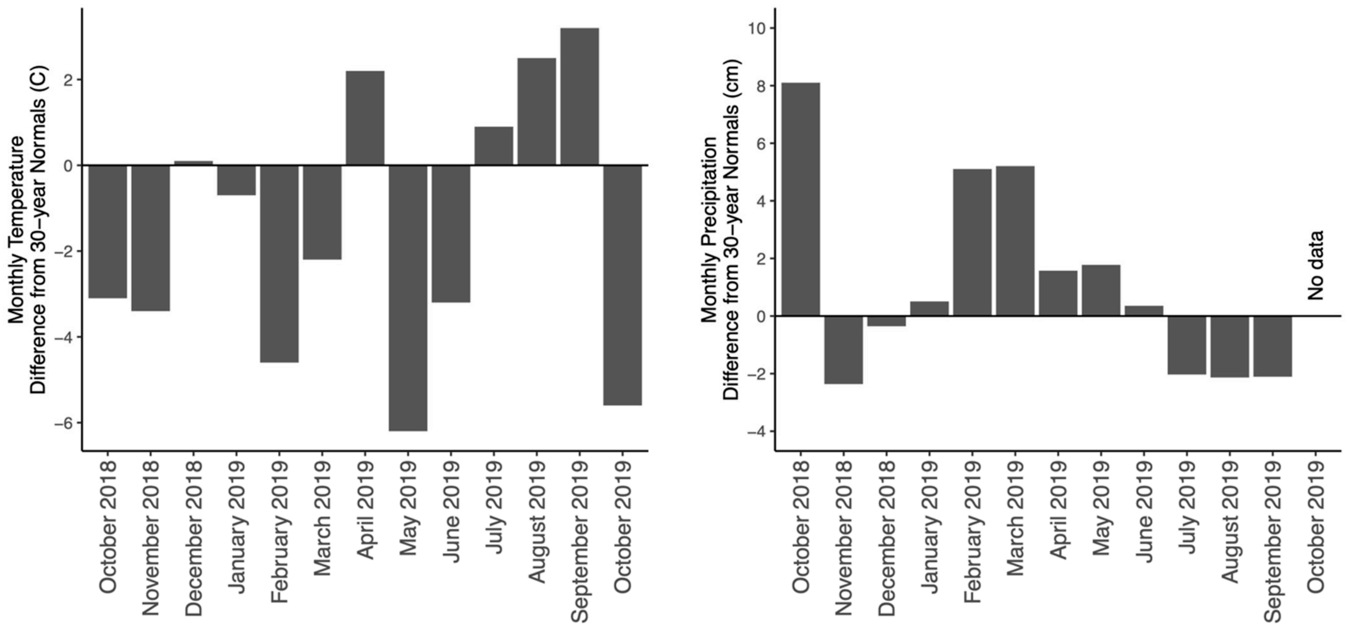

Supplemental Figure S1. Monthly mean temperature and precipitation difference from 30-year normal for the year of cultivation. Climate was cooler and wetter than average (Western Regional Climate Center**,** wrcc.dri.edu, Castle Valley 1SE Station).

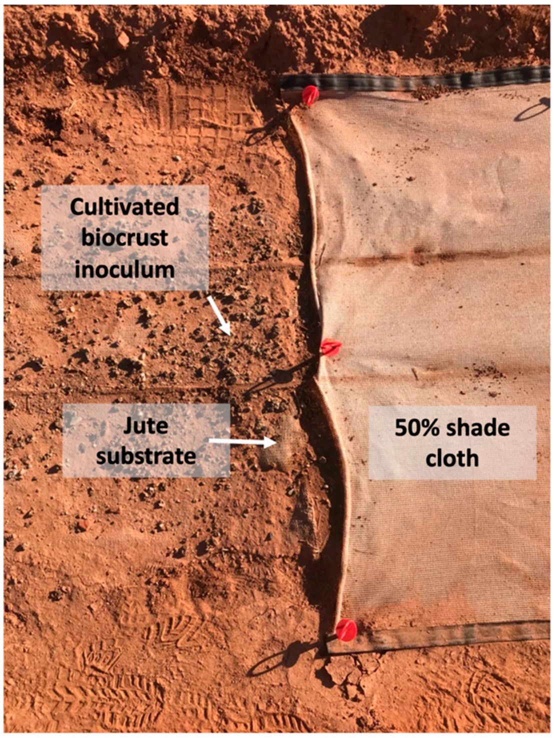

Supplemental Figure S2. Materials used to cultivate biocrust inocula including jute substrate as a layer beneath the inocula and white shade cloth (left). The shade cloth is attached close to the ground with ground staples. Two irrigation lines were laid parallel to the cultivation rows at the center of each row, as shown by the dark indentations in the image.

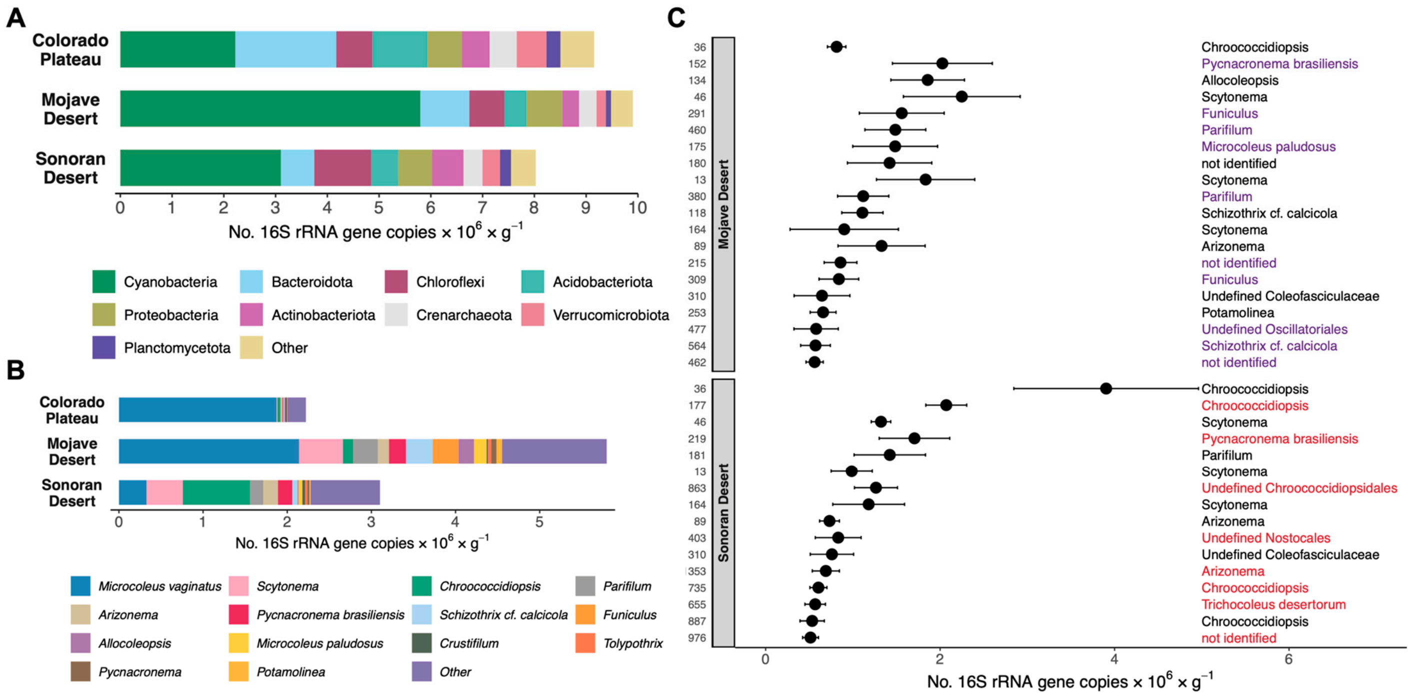

Supplemental Figure S3. A) Phylum-level absolute abundance of reference biocrust communities. B) Genus or species-level absolute abundance of reference biocrust communities. C) Absolute abundance of Mojave and Sonoran cyanobacteria indicator taxa. Genus or species assignments are listed on the right of the figure while ASV numbers are listed to the left of the y-axis. Colors indicate whether the indicator is from the Mojave Desert (purple), the Sonoran Desert (red), or shared (black) indicating presence in both of the hot deserts but not in the Colorado Plateau biocrusts.

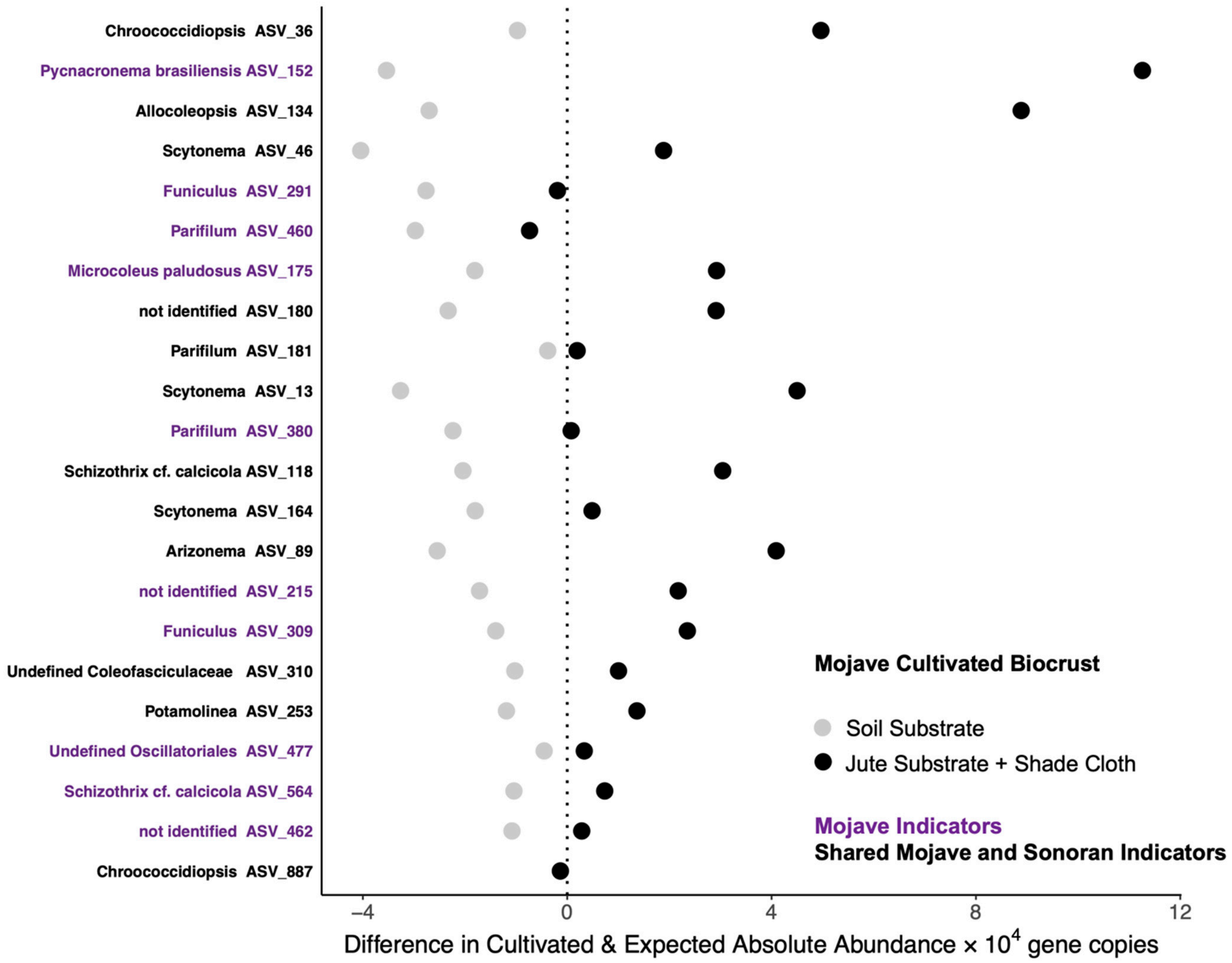

Supplemental Figure S4. Absolute abundance of Mojave cyanobacterial indicator taxa in the inoculation treatment (grey points) and habitat amelioration treatment (black points). Indicator genus and species are provided to the left of the figure, colored by the type of indicator. The x-axis shows the difference in absolute abundance between the cultivated biocrust and the calculated static community. Points to the right of the dotted line are more abundant than expected. Points to the left of the dotted line are less abundant than expected.

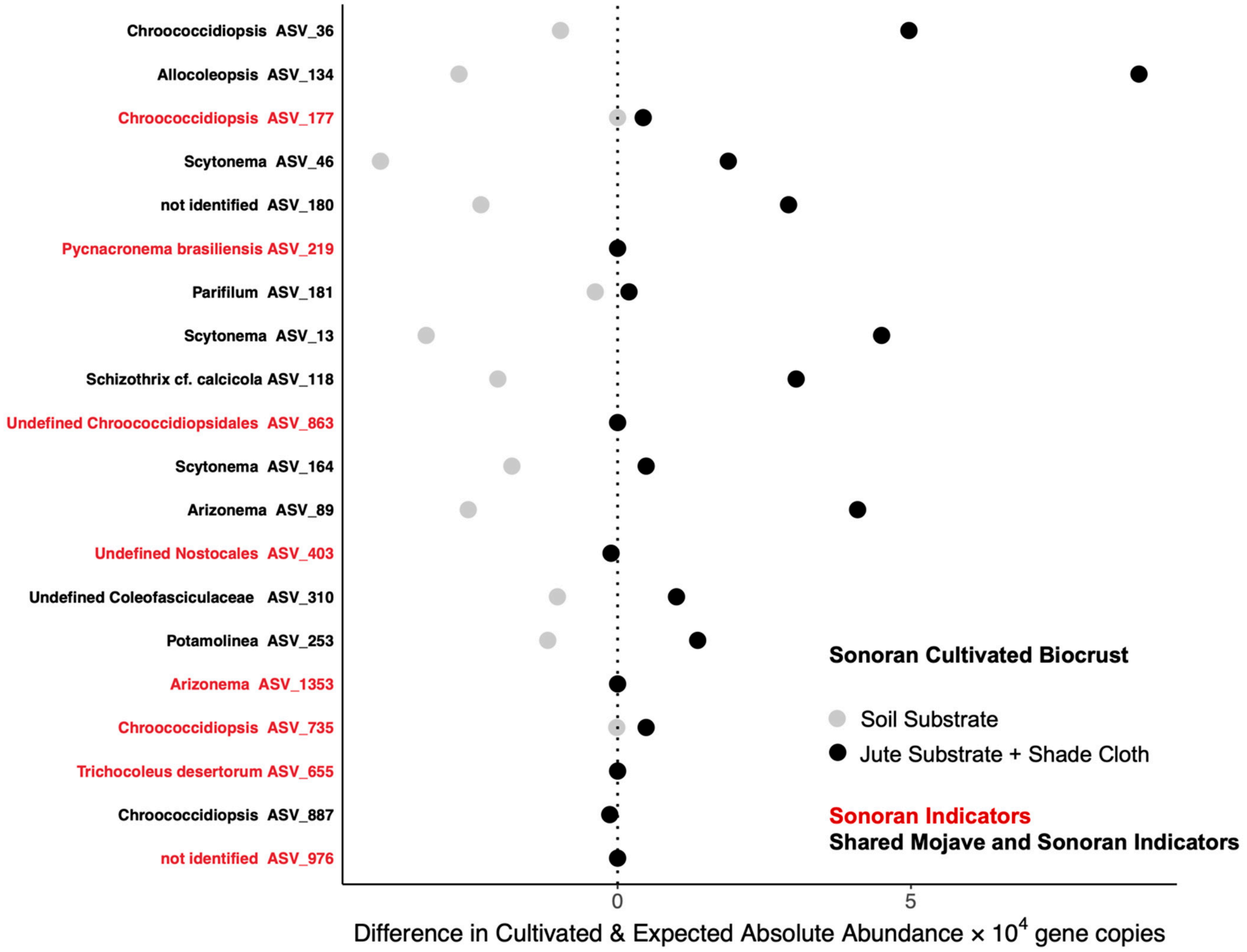

Supplemental Figure S5. Absolute abundance of Sonoran cyanobacterial indicator taxa in the inoculation treatment (grey points) and habitat amelioration treatment (black points). Indicator genus and species are provided to the left of the figure, colored by the type of indicator. The x-axis shows the difference in absolute abundance between the cultivated biocrust and the calculated static community. Points to the right of the dotted line are more abundant than expected. Points to the left of the dotted line are less abundant than expected.

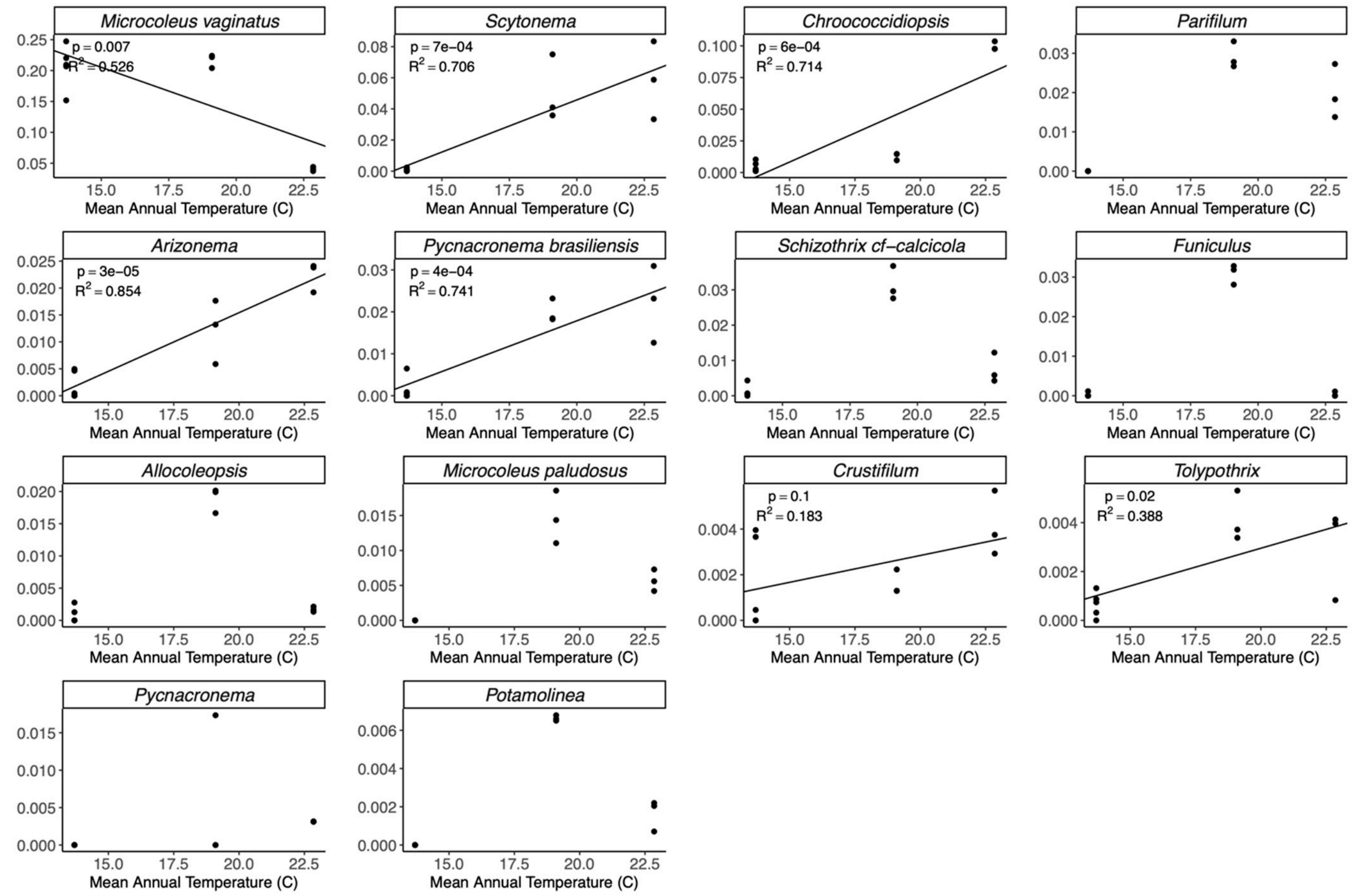

Supplemental Figure S6. Relationship between mean annual temperature (MAT, °C) and the relative abundance of the dominant cyanobacterial genera or species. For those with linear trends, we include a linear model, p-value, and R^2. There were five taxa with significant trends with MAT: *Scytonema* spp., *Chroococcidiopsis* spp., *Arizonema* spp., *Pycnacronema brasiliensis*, *Tolypothrix* spp. Prior work has shown that *Microcoleus vaginatus* abundance has a negative relationship with MAT (Garcia-Pichel et al. 2013) and that *Arizonema* spp. abundance has a positive relationship with MAT (Fernandes et al. 2021). MAT for Colorado Plateau is 13.7°C, for the Mojave Desert is 19.1°C, and for the Sonoran Desert is 22.9°C (Western Regional Climate Center**,** wrcc.dri.edu).
